# Harveian Society

**Published:** 1897-05-08

**Authors:** 


### Medical Progress and Hospital Clinics.

[*The Editor will be glad to receive offers of co-operation and contributions from members of the profession. All letters should be addressed to* The Editor, at the Office, 28 & 29, Southampton Street, Strand, London, W.C.]

### HARVEIAN SOCIETY.

At the last meeting Mr. Rowell read a paper on "The Anæsthetisation of Children," in which, after considering the various means available for the purpose, he described a particular method which he thought possessed great advantages over the use of chloroform as ordinarily employed.

It was well known that the percentage mortality of chloroform in children was much below that in older patients, but he was by no means prepared to admit that symptoms dangerous to life did not occur as often in children as in adults under its use. It was certainly a fact, however, that when such symptoms did arise children were far more amenable to the ordinary methods of restoration than were adults. He thought that when death from chloroform occurred in children it was almost always the result of an overdose. However safe, then, chloroform might be as an anæsthetic for young children, when used with skill by an experienced administrator, the question arose, Was there not a better anæsthetic for routine purposes, and especially for use by those whose experience in administering anæsthetics was not large? The great advantage of ether as an anæsthetic for children lay in the fact that with it there was no risk of failure of the circulation from the action of the drug, and that it was easy to avoid an overdose. The disadvantage was the irritating nature of the ether vapour. If given alone by the open method, one had either to give ether so gradually as to take eight or ten minutes in getting a child ready for operation, or by giving it in larger quantities to risk the production of unpleasant reflexes excited by the irritating vapour—straining, swallowing, coughing, &c.

But having once induced anæsthesia by ether it was much easier to maintain it at its proper depth than was the case with chloroform. Under the latter a condition of quietness and apparent relaxation might be produced which was to a large extent the result of real natural sleep, and this condition was very deceptive; whereas with ether the irritation of the vapour tended to awaken the child, so that when relaxation and absence of the reflexes interfering with regular breathing were once attained there was a true anæsthesia.

One way of avoiding the preliminary difficulties in the administration of ether was the production of a preliminary anæsthesia by means of gas, and for those who were much engaged in this sort of work the gas and ether method would always be valued, and, speaking generally, it might be said that this was the plan for those above five or six years of age. But the method which he wished to recommend in the case of young children was the administration of the A.C.E. mixture, followed by that of pure ether by the open method. This was also suitable up to the ages of 12 or 14, where the administrator was not experienced in the use of gas and ether in such patients, and it was to be preferred to the latter in children of any age in whom any obstruction of the upper air channels existed.

The apparatus required was a Skinner's mask, or for smaller children a mask made from the corner of a towel, and a Rendle's cone with two sponges.

The patient should lie if possible in the lateral position, or, at any rate, with the head slightly turned to one side, and only a little raised. A.C.E. was to be added to the Skinner from a drop-bottle gradually, so that beginning with a drop at a time the whole surface became wetted by the end of one minute. The rapidity with which this could be done depended mainly upon the character of the breathing. If the vapour caused embarrassment of breathing or much swallowing one proceeded more gradually; while, on the other hand, if there were much crying and breathing were free, anæsthetisation proceeded with much greater rapidity, and in this case the inhaler should be removed from the face for two complete full inspirations of air as soon as the crying ceased. Should the breathing become embarrassed after crying had ceased, or should there be much swallowing, this would indicate that the vapour was too strong, and the inhaler should then be removed an inch or two from the face. Keeping the inhaler visibly wet, at the end of about half a minute longer one would find that the breathing became fairly free, and when that was the case the Rendle mask, on the sponge in which a half to two drachms of A.C.E. had just been poured, was rapidly substituted. This gave a considerably stronger vapour than had been breathed before, and it might cause a little straining and swallowing, and might even wake the child up and make it cry, but by removing the cone a little further from the face (never quite withdrawing it) such difficulty would soon be overcome, and so long as the breathing continued good in frequency and volume, and not too much interrupted by swallowing or coughing, the mask could be kept fairly closely applied to the child's face. After about a minute and a quarter or a minute and a half from the change of mask, the child would breathe this vapour freely and regularly, and when this happened, one to three drachms of ether were to be poured upon the sponge. This should be done rapidly, the child only obtaining about one breath of air during the change. Again the stronger vapour might slightly awake the child, but very soon it would breathe the vapour as if it were ordinary air, and when this happened, two, three, or even four drachms of ether should be poured on a fresh sponge, which should be substituted for the one which had contained the A.C.E. From this time the child would be taking only ether. If the vapour given off were very strong, the child might again be somewhat roused by it, but in a very little time—eight or ten breaths—the breathing would become free, and the child would then be ready for any operation. The point to be remembered was that the larynx was so susceptible to ether vapour that whenever a child could breathe strong ether vapour without swallowing, coughing, or a catch or spasm in its respiration, that child was anæsthetic for surgical purposes.

Anæsthesia once produced could be easily kept up by alternately applying the mask and leaving it off for a few respirations; keeping it applied more continuously if the patient was unable to breathe the strong vapour easily, and always removing it if it was found already able to do so. The condition of the laryngeal reflexes was the test of the anæsthesia, and by withholding the ether whenever it was found to be easily taken, all chance of over-dose was avoided.

In the case of very fractious patients the Skinner's mask was dispensed with, and the administration commenced from the Rendle. The after effects of the method were stated to be trivial, and any risk of bronchitis almost certainly preventable by avoidance of exposure to cold for a few hours afterwards. The advantages claimed for the method were the short period of induction, the complete freedom from movement during the operation which is attained, the certainty of knowledge possible as to the degree of narcosis present at any particular moment, and the absence of danger.

